# Dispersal into the Qinghai–Tibet plateau: evidence from the genetic structure and demography of the alpine plant *Triosteum pinnatifidum*

**DOI:** 10.7717/peerj.12754

**Published:** 2022-02-01

**Authors:** Hai Rui Liu, Gulzar Khan, Qingbo Gao, Faqi Zhang, Wenhui Liu, Yingfang Wang, Jie Fang, Shilong Chen, Sahib Gul Afridi

**Affiliations:** 1 State Key Laboratory of Plateau Ecology and Agriculture, Qinghai University, Xining, Qinghai Province, China; 2 College of Eco-Environmental Engineering, Qinghai University, Xining, Qinghai Province, China; 3 Key Laboratory of Adaptation and Evolution of Plateau Biota, Northwest Institute of Plateau Biology, Chinese Academy of Sciences, Xining, Qinghai Province, China; 4 Institute for Biology and Environmental Sciences, Carl von Ossietzky University Oldenburg, Oldenburg, Lower Saxony, Germany; 5 Department of Geological Engineering, Qinghai University, Xining, Qinghai Province, China; 6 Department of Biochemistry, Abdul Wali Khan University Mardan, Marden, Khyber-Pakhtunkhwa, Pakistan

**Keywords:** Genetic diversity, Alpine plants, Palaeodistributional modelling, Range shifts

## Abstract

*Triosteum pinnatifidum* Maxim., an alpine plant, is traditionally used for several medicinal purposes. Here, both chloroplast DNA sequences and nuclear low copy sequence markers were used to investigate the genetic diversity and population structure of *T. pinnatifidum*. Materials were collected from thirteen localities in the northeast Qinghai–Tibet Plateau (QTP) and adjacent highlands and advanced analytical toolkits were used to access their origin and range shifts. The results revealed a higher level of population differentiation based on chloroplast DNA (cpDNA) concatenated sequences compared with the nuclear DNA sequences (*F*_ST_ = 0.654 for cpDNA, *F*_ST_ = 0.398 for AT103), indicating that pollen flow was still extensive in *T. pinnatifidum*. A decline in haplotype variation was observed from the plateau edge and adjoining highlands toward the platform of the QTP. The hypothesis “dispersal into the QTP,” proposing that *T. pinnatifidum* experienced migration from the plateau edge and adjacent highlands to the platform, was supported. These results were in line with the hypothesis that multiple refugia exist on the plateau edge and adjacent highlands rather than on the plateau platform. Our unimodal mismatch distribution, star-like network supported a recent expansion in *T. pinnatifidum*.

## Introduction

Fluctuations in the environment and climate have a great effect on both genetic structure and species distribution (Hewitt, 2000; Meng et al., 2007; De Dato et al., 2020). Quaternary climatic oscillations and environmental changes on the Qinghai–Tibet Plateau (QTP) since 2.6 million years ago (Mya) had a great effect on species range expansion shifts and speciation in the QTP (Zhang et al., 2005; Meng et al., 2007; Jia et al., 2012; Gengji et al., 2018; Li et al., 2020). At the same time, due to QTP uplift and intensified East Asian monsoons, greater changes in climate may have occurred between glacial and inter-glacial periods (Jia et al., 2012; Lu et al., 2014; Gao et al., 2016). In addition, geological reports suggest that the central plateau reached an average height of 4–5 km 40 Mya, and the southern, western, and northern mountains around the QTP reached their present altitude 8 Mya (Wang et al., 2014a; Wang et al., 2014b; Renner, 2016; Khan et al., 2018a; Khan et al., 2018b; Su et al., 2019). Therefore, the effect of plateau uplift on the genetic diversity of species was presumably not as large as expected. However, there are reports that the change in climate, topography, and environment accompanied the uplift of the plateau and triggered the fragmentation of species’ ranges and geographical distribution (Gengji et al., 2018; Jia et al., 2020). Similarly, the glacial and interglacial cycles strengthened climate and environment fluctuations creating conditions for new niches and the formation of plant species (Meng et al., 2007; Liu et al., 2012; Lu et al., 2014).

The QTP is the youngest and highest plateau in the world, with an average altitude of more than 4,500 m and an area of 2.5 × 106 km^2^ (Shi et al., 1999). The QTP and adjacent Qinling and Taihang mountain ranges are characterized by unique topographic and environmental conditions with several residing biomes (*e.g.*, alpine tundra, meadow, and montane forests) (Ni & Herzschuh, 2011). Such conditions provide excellent habitats for biota, and some served as refugia during severe environmental episodes particularly during glaciation and also played the role of diversification centers during post-glaciation (Liu, Wang & Huang, 2009; Zhang, Volis & Sun, 2010; Jia et al., 2012; Favre et al., 2016). Thus, this region is considered a precious gene resource center for many plants with latent economic value and ecological significance. Similarly, this region continuously provides valuable genes to benefit other species, including humans (Wang, Yang & Liu, 2005; Lu et al., 2015; Wu et al., 2016).

Extreme environments, including glaciers, can threaten the survival of plants and cause a sharp reduction in plant distribution to refuge areas (Wang et al., 2009a; Gao et al., 2016; De Dato et al., 2020). However, some species expand their ranges during interglacial periods and colonize new habitats (Hewitt, 2000; Zhang et al., 2005; Meng et al., 2007; Zhao et al., 2019). In refugia, species are relatively less influenced by the environment during glaciation and thus contain higher levels of genetic diversity than other recently occupied areas. There is a consensus that refugia are formed under the shelter of high mountains and valleys during glaciation (Zhang et al., 2005). In addition, refugia contain ancient haplotypes and more endemic haplotypes (Ma, 2012; Zhou et al., 2012; Lu et al., 2014; Khan et al., 2018a; Khan et al., 2018b; Päckert et al., 2020).

Glacier refugia are found on several mountains in China (Liu et al., 2012; Zhao et al., 2019). However, research on the distribution of glacial refugia on the QTP and adjacent highlands is still insufficient. There are different hypotheses explaining the species range shifts on the QTP (Muellner-Riehl, 2019). According to the first hypothesis, microrefugia exist for several species, which means that the species have shifted on small scales rather than on a large range with climate change (Gao et al., 2016; Gengji et al., 2018). However, the alternative hypothesis is that the refugia are in the QTP platform, and dominant vegetation recolonized deglaciated regions around refugia (Gao et al., 2016). This hypothesis is indicated by many studies, such as studies on *Aconitum gymnandrum* (Wang et al., 2009a) and *Potentilla glabra (Wang et al., 2009b*).

The third hypothesis suggests that species retracted to one or more shelters at the plateau edge during the cold period and expanded during inter-glaciation to the central plateau. Studies on *Picea crassifolia* (Meng et al., 2007), *Rana kukunoris* (Zhou et al., 2012), and *Spiraea alpine* (Khan et al., 2014) support that multiple refugia occurred on the northern and northeastern edge of the QTP.

Investigations on the molecular diversity and biogeographic structure of alpine plants in the QTP and adjoining highlands have greatly increased in recent years (Wen et al., 2014; Ebersbach et al., 2017; Liu, 2019); however, with the advent of new analytical tools and the ease of generating molecular data, more research in the QTP is needed to robustly support the hypotheses regarding demographic range shifts. Here, *Triosteum pinnatifidum* was used as a biological model to substantiate the hypotheses of species range shifts distributed on the QTP. *T. pinnatifidum* (Caprifoliaceae) is a perennial species distributed in sunny places along the streamside and coniferous forests from 1,800 to 2,900 m (Yang et al., 2011).

This species is very well known in traditional Chinese medicine, where their roots are called “Tian Wang Qi” and used to treat rheumatic lumbocrural pain, bruises, dyspepsia, and menoxenia (Guo et al., 2003). The chemical constituents of both root and aerial parts of *T. pinnatifudum* have been reported to contain many biologically active compounds, including loganic acid, iridoids, monoterpene indole alkaloids, and monoterpene diglycoside (Chai et al., 2010; Ding et al., 2013; Chai et al., 2014; Huang et al., 2014). *Triosteum* is an entomophilous plant genus (Yang et al., 2011; Zhou, 2012). Even the seed dispersal pattern of *T. pinnatifidum* is still unknown. Compared with seeds of other species from the family Caprifoliaceae, the seeds of *T. pinnatifidum* are larger and heavier, and this could impact seed dispersal by wind and gravity (Jacobs, Lens & Smets, 2009). In addition, in germination experiments, the hard and robust endocarp of *T. pinnatifidum* limited the germination rate and influenced dispersal efficiency. There was also a study on the morphology and histology of *T. pinnatifidum* (Cao, Li & Wu, 2014); however, there is no research regarding its genetic diversity and population structure on the QTP and adjoining highlands. As the chloroplast DNA is maternally inherited in most angiosperms, and no research denied the maternal inheritance of chloroplast DNA for *Triosteum*. Thus, cp DNA of *T. pinnatifidum* was assumed as matrilineal inheritance in our study.

Our objectives were (i) to explore the molecular diversity and population structure using both maternally inherited chloroplast DNA and biparentally inherited nuclear DNA sequences, and (ii) to investigate distribution range shifts (to the plateau) of *T. pinnatifidum* using phylogeography and ecological niche modeling. To this end, sequences of two chloroplast DNA fragments (*rbc*L-*acc*D and *rps*15-*ycf*1) and one nuclear DNA low copy sequence (AT103) were generated for 13 localities and analyzed with advanced toolkits. For demographic history analysis, palaeodistributional modeling was used. To clarify our objectives, all localities were divided into two groups: the Edge group (P7–P13) and the Platform group (P1–P6).

## Materials & Methods

### Sampling

**Figure 1:**
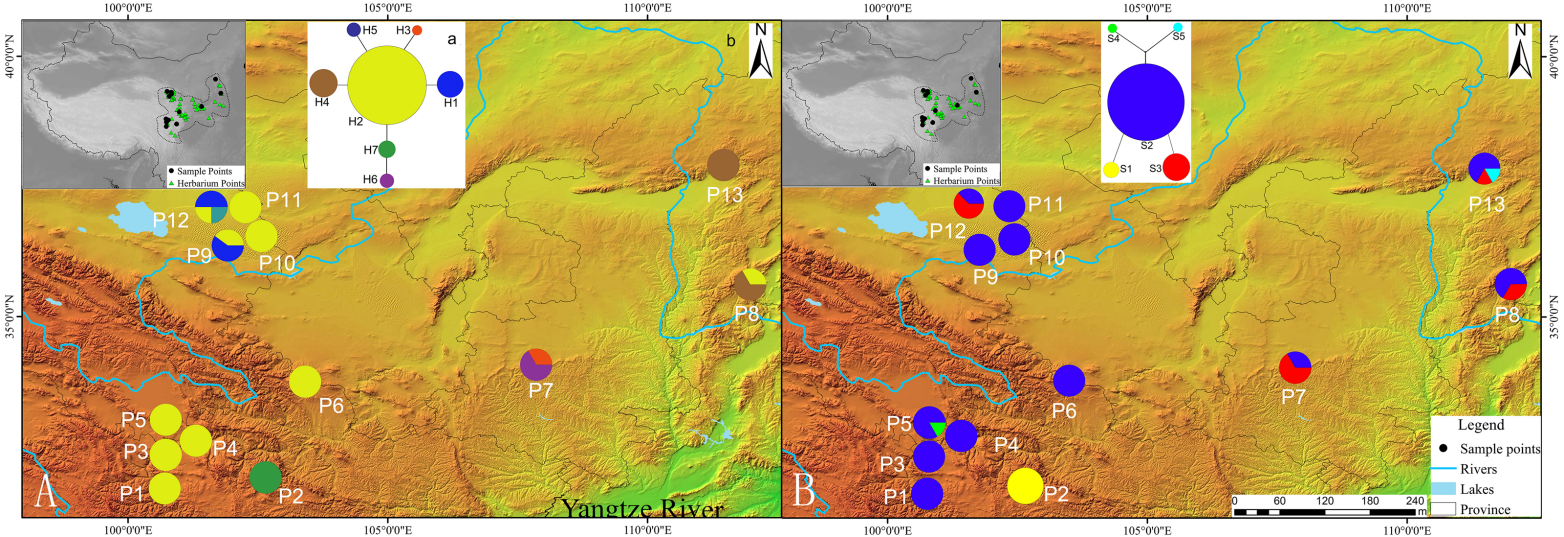
Geographic distribution of cpDNA and AT103 haplotypes detected among 13 populations of *Triosteum pinnatifidum*, (A) cpDNA, (B) AT103. Pie charts show the proportions of haplotypes within each population.

Different field expeditions were carried out during 2008 and 2017 to collect as many of the locality as possible in accordance with the specimen records in the herbaria. From the field, fresh leaves of 3–14 individuals were collected for each locality, separated by at least 20 m. The distance between localities was usually more than 50 km. A total of 86 individuals from 13 localities were collected in the Sichuan, Qinghai, Shaanxi, and Shanxi provinces. Among these localities, five were from the QTP, three localities were from the adjoining highlands region, and five localities were from the plateau edge (Table 1 and Fig. 1). The leaf materials were dried in silica gel and stored at room temperature. Voucher specimens of the localities were deposited in the Herbarium of the Northwest Institute of Plateau Biology (HNWP), Xining, Qinghai, China.

**Table 1:** Sample data and haplotypes of *Triosteum pinnatifidum*. For each locality, haplotype composition (H1–H7 for cpDNA, S1–S5 for AT103) is indicated. SC, Sichuan; QH, Qinghai.

**Code**	**Sample location**	**Voucher specimens**	**Latitude(N)**	**Longtitude(E)**	**Altitude (m)**	**Number**	**cpDNA** **haplotypes**	**AT103 haplotypes**
P1	Luhuo, SC	Chensl1890	N31°37′29.0″	E100°43′12.0″	3,470	14	H2(14)	S2(14)
P2	Hongyuan, SC	Chensl1842	N31°50′15.3″	E102°41′11.4″	3,370	3	H7(3)	S1(3)
P3	Rangtang, SC	Chensl-1162	N32°19′16″	E100°49′50″	3,430	6	H2(6)	S2(6)
P4	Aba, SC	Chensl-1178	N32°35′55″	E101°14′26″	3,090	4	H2(4)	S2(4)
P5	Banma, QH	Chensl-0335	N32°48′46″	E100°49′08″	3,600	6	H2(6)	S2(5) S4(1)
P6	Ruoergai, SC	Chen2012028	N33°40′50.6″	E103°27′49.1″	3,150	14	H2(14)	S2(14)
P7	Taibaishan, Shaanxi	Zhang2015005	N34°00′40″	E107°48′34″	2,863	3	H3(1) H6(2)	S2(1) S3(2)
P8	Lishan, Shanxi	Zhang2015032	N35°30′46″	E111°56′02″	1,792	3	H2(1) H4(2)	S2(2) S3(1)
P9	Pingan, QH	Chensl-1759	N36°20′24.0″	E101°54′58.5″	2,690	5	H1(3) H2(2)	S2(5)
P10	Ledu, QH	Chensl-1820	N36°41′16.9″	E102°24′09.3″	2,650	3	H2(3)	S2(3)
P11	Huzhu, QH	Chensl-1725	N36°55′07.5″	E102°22′01.1″	2,690	11	H2(11)	S2(11)
P12	Datong, QH	Liu2017001	N37°50′45″	E111°28′02″	2,750	8	H1(4) H2(2) H5(2)	S2(3) S3(5)
P13	Jiaocheng,Shanxi	Zhang2015051	N37°50′45″	E111°28′02″	1,823	6	H4(6)	S2(4) S3(1) S5(1)

### Laboratory protocols and sequencing

Genomic DNA was extracted from the silica-gel dried leaves following a modified Cetyltrimethyl Ammonium Bromide (CTAB) protocol (Doyle & Doyle, 1987). The quality and quantity of the genomic DNA was assessed using a spectrophotometer (Nanodrop™; Thermo Scientific, Waltham, MA, USA) and gel electrophoresis. Both the chloroplast DNA and nuclear regions were amplified using the primers described by Li et al. (2008) and Dong et al. (2012).

Polymerase chain reactions (PCR) were performed at a 50 µL volume containing 1.2 µL template DNA (∼20 ng), 5 µL 10 × PCR buffer (with Mg^2+^), 2.0 µL 10 mM dNTPs, 1 µL 5 pM of each primer, and 0.4 µL (1.5 units) Taq polymerase (Takara, Dalian, China). The amplification conditions were 5 min at 95 °C, followed by 35 cycles of 50 s at 95 °C, 1 min at 58 °C, and 1 min at 72 °C, with a final extension of 6 min at 72 °C. PCR products were purified using the CASpure PCR Purification Kit (CASarry, Shanghai, China) following the manufacturer’s protocols. Sequencing reactions and analysis were performed using an ABI 3730xl DNA sequencer (Applied Biosystems, Foster City, CA, USA).

The chromatograms of each sequence were checked visually using Chromas ver. 2.33 (http://www.technelysium.com.au). The DNA sequences were aligned in Clustal_X (Thompson, 1997) and then checked by visual inspection to remove any bias introduced during sequencing. To retrieve the haplotypes, DnaSP ver. 5.0 was used (Librado & Rozas, 2009). All newly generated sequences from *T. pinnatifidum* were submitted to GenBank (MH277369 –MH277382). The cpDNA fragments, *rbc*L-*acc*D and *rps*15-*ycf*1, were concatenated for downstream analyses.

### Genetic diversity and population structure

Two different analyses were performed to detect the genetic structure and phylogenetic relationships among haplotypes in *T. pinnatifidum* for both the cpDNA and nDNA haplotypes. A haplotype network was constructed using the median-joining method and maximum parsimony (MP) calculations as implemented in Network ver. 5.0.0.3 (Bandelt, Forster & Rohl, 1999; Polzin & Daneshmand, 2003). To supplement the Network results, the genetic groups and spatial genetic uniqueness for *T. pinnatifidum* were further elaborated with the SAMOVA program ver. 1.0 (Dupanloup, Schneider & Excoffier, 2002). SAMOVA was used to define groups of localities that were geographically homogeneous and maximally differentiated from each other. The number of initial conditions for this analysis was set to 100, and pairwise differences (DNA) were chosen as the molecular distance.

After confirming the genetic groups, the average gene diversity within localities (*H*_S_), total gene diversity (*H*_T_), *G*_ST_ (Nei, 1987), and *N*_ST_ (Grivet & Petit, 2002) were estamated for the overall localities and two groups as employed in PERMUT ver. 2.0 (De Meulemeester et al., 1996). *G*_ST_ and *N*_ST_ were also used to estimate the population differentiations; *G*_ST_ only considers haplotype frequency, whereas *N*_ST_ also considers sequence similarities between haplotypes. A permutation test with 1,000 permutations was used to compare *G*_ST_ and *N*_ST_. Significantly higher values of *N*_ST_ compared with the estimated *G*_ST_ indicated the presence of a phylogeographical structure (De Meulemeester et al., 1996).

ARLEQUIN version 3.11 (Excoffier, Laval & Schneider, 2005) was used to conduct an analysis of molecular variance (AMOVA) for the overall localities and two groups of *T. pinnatifidum*. This analysis was further extended to calculate the F-statistic (*F*_ST_). The significance of *F*_ST_ was tested using 1,023 permutations. Formula Nm = (1−*F*_ST_)/(4*F_ST_) was used to estimate the gene flow for overall localities (Slatkin & Barton, 1989).

### Range shifts

Genetic and distributional modeling toolkits were used to investigate the demographic history of *T. pinnatifidum*. From the genetics toolkits, Tajima’s *D* and Fu’s *Fs* neutrality tests were performed with 1,000 simulated samples using ARLEQUIN ver. 3.11 (Choi et al., 1995). In favor of recent range expansion, negative values are expected when there are recent population expansions (Li, Morzycka-Wroblewska & Desiderio, 1989). Similarly, unimodal distributions reflect rapid growth from small populations, while multimodal distributions reflect long-term population stability (Slatkin & Hudson, 1991; Rogers & Harpending, 1992).

The sum of squared deviation (SSD) was used as a statistical test to accept or reject the hypothesis of sudden population expansion. Harpending’s raggedness index (HRI) (Jones, 1994) and its significance were calculated to quantify the smoothness of the observed mismatch distribution. SSD and HRI test were conducted using ARLEQUIN ver. 3.11. Additionally, the pairwise mismatch distribution (MDA) was analyzed as suggested by Rogers & Harpending (1992), for the overall localities and each group of datasets assuming past exponential growth and historical population stasis using DnaSP ver. 5.0 (Librado & Rozas, 2009).

For the paleodistribution analysis, the occurrence points were noted during the field visits from 2008 to 2017 for the entire distribution. Their distribution also searched for available occurrences from the Global Biodiversity Information Facility (GBIF, http://www.gbif.org/) and the occurrences were grouped. After this, the best ecological niches were fitted in the present and past (Mid-Holocene, 21 kya [LGM], and 120 kya [LIG]) using different available algorithms: surface range envelope (SRE; Csuti, Margules & Austin, 1992), generalized boosting models (GBM; or boosting regression trees, BRT; Friedman, 2001), generalized linear models (GLM; Peter & John, 1989), Classification Tree Analysis (CTA; Breiman, 2001), Artificial Neural Network (ANN; Ripley, 1996)*,* Flexible Discriminant Analysis (FDA; Hastie, Tibshirani & Buja, 1994), MARS (Friedman, 1991), Random Forests (RF; Breiman, 2001), and MAXENT (Phillips, Dudík & Schapire, 2004).

All algorithms were employed in BIOMOD2 (https://rforge.r-project.org/R/?group_id=302) within an R environment through different packages. Briefly, six bioclimatic variables (annual mean temperature; mean diurnal range (mean of monthly {max temp-min temp}); isothermality; annual precipitation; precipitation of driest; and precipitation seasonality (coefficient of variation), Fig. S1) were selected, showing low correlation and high informativeness after a jackknife procedure on the 19 BIOCLIM data downloaded from WorldClim (Hijmans et al., 2005). The success of all the models was assessed using true skill statistics (TSS) and area under the curve (AUC, curve-receiver operating characteristic curve) values of > 0.7. To identify suitable areas for each period, the data were delimited for the People’s Republic of China region with 2.5 arc-minutes.

To describe patterns of *T. pinnatifidum* effective population changes over time and obtain date estimates of major demographic events, the BDSKY package in Beast version 2.6.6 (Bouckaert et al., 2019) was used for Bayesian Skyline Plot (BSP) analysis. The cp genes sequences of all individuals from 14 localities were compared using MAFFT (Katoh & Standley, 2013) software in Phylosuite (Zhang et al., 2020), A GTR model was used, as seleceted by ModelFinder (Kalyaanamoorthy et al., 2017). Strict clock was used and the clock rate was set to at 3 × 10^−9^ s s/y (synonymous substitution per years; (Wolfe, Li & Sharp, 1987)). The MCMC analyses were run for 5 ×108 steps and sampling every 1000 steps. The TRACER version 1.7.2 (Rambaut & Drummond, 2007) was used to analyses the log files and tree files, to verify that the analyses was ran properly and the results were consistent across runs. Bayes factor analyses were conducted to assess whether the Bayesian skyline model was statistically better than a simpler demographic model of constant population size using the same parameter settings.

## Results

### Genetic diversity

A total of seven chloroplast DNA (cpDNA) haplotypes (H1–H7) and five AT103 haplotypes (678 pb; S1–S5) were retrieved based on polymorphic sites across the concatenated cpDNA sequences (1,373 pb; Table 1). In cpDNA haplotypes, four unique haplotypes (private haplotypes) were identified, while in AT103, private haplotypes were found. AMOVA for the cpDNA revealed higher population differentiation: 65.4% of the total genetic variation occurred among localities, compared with 34.6% within localities. However, the nuclear DNA-based haplotypes demonstrated a higher level of within-locality variation (60.23%) compared with among-locality variation (39.77%), revealing lower population differentiation (Table 2). Similar variation patterns were observed in the edge group for the two datasets. In the platform group, all genetic variation in cpDNA was among localities, while 66% of AT103 variation was among localities (Table 3). The values of total genetic diversity (*H*_T_) were much higher than those of gene diversity within localities (*H*_S_) for *T. pinnatifidum* in the overall localities based on the two datasets (*H*_T_ = 0.204, *H*_S_ = 0.626 for cpDNA; *H*_T_ = 0.216, *H*_S_ = 0.417 for AT103), the edge group (*H*_T_ = 0.378, *H*_S_ = 0.798 for cpDNA; *H*_T_ = 0.362, *H*_S_ = 0.590 for AT103), and the platform group (*H*_T_ = 0.000, *H*_S_ = 0.333 for cpDNA; *H*_T_ = 0.056, *H*_S_ = 0.378 for AT103; Table 4). The dominant haplotypes (H2 for cpDNA and S2 for AT103) were shared by most localities; only localities P2 shared no common haplotypes with other localities for both the cpDNA and AT103 datasets.

**Table 2:** Analysis of molecular variance (AMOVA) of AT103 haplotypes for localities of *Triosteum pinnatifidum*.

**Regions**	**Source variation**	**d.f.**	**SS**	**VC**	**Variation (%)**	***F***_**ST**_
Whole distribution	Among localities	12	7.80	0.08	39.77	
	Within localities	73	9.04	0.12	60.23	
	Total	85	16.84	0.20		0.397
Edge group	Among localities	6	3.30	0.06	24.11	
	Within localities	32	6.54	0.20	75.89	
	Total	38	9.84	0.26		0.24^*^
Platform group	Among localities	5	3.10	0.08	66.06	
	Within localities	41	1.67	0.04	33.94	
	Total	46	4.77	0.12		0.66^*^
Edge group versus Platform group	Among groups	1	1.24	0.01	5.67	
	Among localities within groups	11	6.40	0.08	37.84	
	Within localities	73	8.21	0.11	56.49	
	Total	85	15.85	0.20		0.44^*^

**Notes.**

d.f. degree of freedom SS sum of squares VC variance component

**Table 3:** Analysis of molecular variance (AMOVA) of cpDNA haplotypes for localities of *Triosteum pinnatifidum*.

**Regions**	**Source variation**	**d.f.**	**SS**	**VC**	**Variation (%)**	***F***_**ST**_
Whole distribution	Among localities	12	15.92	0.19	65.40	
	Within localities	73	7.37	0.10	34.60	
	Total	85	23.29	0.29		0.65^*^
Edge group	Among localities	6	11.40	0.31	57.63	
	Within localities	32	7.37	0.23	42.37	
	Total	38	18.77	0.54		0.58^*^
Platform group	Among localities	5	2.81	0.08	100	
	Within localities	41	0.00	0.00	0	
	Total	46	2.81	0.08		1.00^*^
Edge group versus Platform group	Among groups	1	1.71	−0.00046	−0.16	
	Among localities within groups	11	14.21	0.19	65.53	
	Within localities	73	7.37	0.10	34.63	
	Total	85	23.29	0.29		0.65^*^

**Notes.**

d.f. degree of freedom SS sum of squares VC variance component

### Population structure

The private haplotypes and localities with higher variation were mainly located on the very margin of the QTP or adjoining highlands (Fig. 1). The SAMOVA grouping strategy failed to detect any meaningful geographical groups in the sampled localities. When the K values were increased from 2, each newly defined group was represented by a single locality that included private haplotypes or higher genetic variation, suggesting a weak phylogeographic structure. Due to the distribution ecoregions of sample locations and the genetic pattern of *T. pinnatifidum*, all localities were divided two groups: the Edge group (P7–P13) and the Platform group (P1–P6). The grouping strategy was organized by the: (1) difference in the altitude of localities, with the Edge group under 2,900 m and the Platform group above 3,000 m (Table 1); (2) location, with Platform group belonging to the northern part of Hengduan mountain, the Edge group P9–P12 belonging to the Qianlian mountain and the remainings located beyond the QTP (Zhang, Li & Zheng, 2002; Fig. 1). There was an environmental distinction between the two groups.The weak phylogeographic structure was confirmed by haplotype-identification permutation tests (*N*_ST_ < *G*_ST_). For both cpDNA and AT103 haplotypes, *N*_ST_ (number of substitution types) was smaller than *G*_ST_ (inter-locality differentiation) overall and in the two groups (except for AT103 in Edge group), and a nonsignificant phylogeographic structure was shown (Table 4).

Our network analysis indicated that the cpDNA haplotype H2 and AT103 haplotype S2 could be the origin of other haplotypes because they remained at the center of the structure (Fig. 1). They were the most widely distributed and most frequent localities in the *T. pinnatifidum* Northwest China distribution. Other young haplotypes surrounded these two haplotypes as branches in the network. The unique cpDNA haplotype H3 and idiotypic AT103 haplotype S5 were only distributed in adjacent highlands (Shaanxi and Shanxi provinces). Another unique AT103 haplotype S4 only existed on the QTP platform. Additionally, the haplotype network was star-like, indicating the expansion of *T. pinnatifidum* localities.

### Demographic history

**Figure 2:**
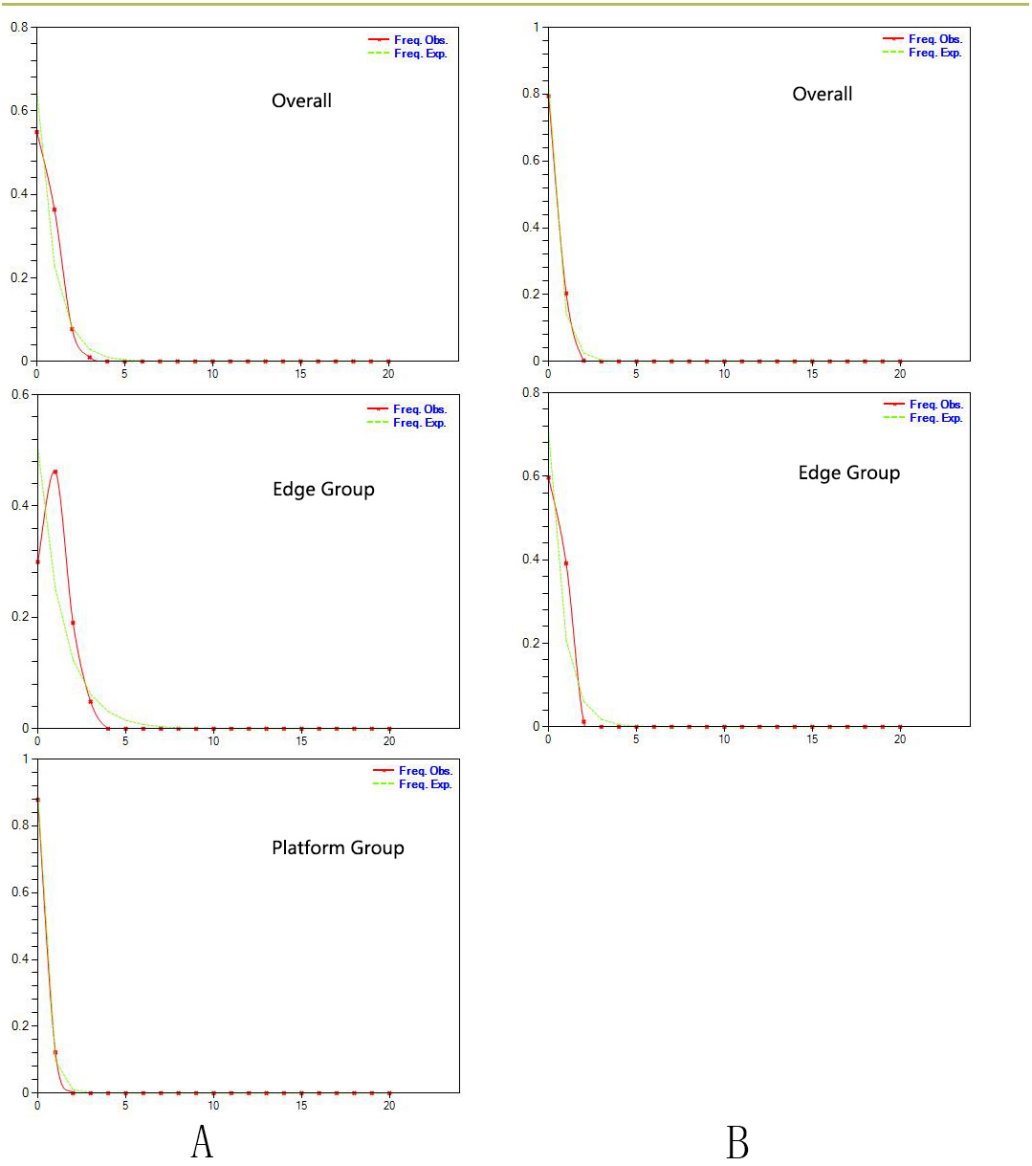
(A) Mismatch distribution for chloroplast DNA sequence data of *Triosteum pinnatifidum*. (B) Mismatch distribution for nuclear DNA sequence data of *T. pinnatifidum*. Solid lines represent expected differences among sequences, whereas dashed lines are drawn from the observed differences.

Tajima’s *D* values based on chloroplast DNA and AT103 sequences overall and in the two groups were negative or zero but not significant (−1.25 overall, −0.82 for Edge group, and −0.63 for the Platform group). Fu’s *Fs* statistics for cpDNA also showed nonsignificant negative or positive values for the two datasets, which revealed the relative stability of the *T. pinnatifidum* localities (Table 5). However, mismatch distributions of both datasets overall and in the two groups were unimodal, indicating recent population expansion (Fig. 2). Due to insufficient polymorphisms, the mismatch distribution of AT103 in the Platform group could not be computed. The values of the sum of squared deviations (SSD) and the raggedness index based on both datasets were not significant (cpDNA: *P*_SSD_ = 0.38 *vs.* 0.15 *vs.* 0.13, *P*_HRI_ = 0.32 *vs.* 0.09 *vs.* 0.43; AT103: *P*_SSD_ = 0.41 *vs.* 0.19 *vs.* 0.36, *P*_HRI_ = 0.56 *vs.* 0.42 *vs.* 0.65 for both (overall), Edge, and Platform groups, respectively; Table 5).

**Table 4:** Estimates of H_*S*_, H_*T*_, G_*ST*_, N_*ST*_ for cpDNA and AT103 haplotypes of *Triosteum pinnatifidum*.

**Sequences**	**Groups**	***H***_**S**_	***H***_**T**_	***G***_**ST**_	***N***_**ST**_
cpDNA	Overall localities	0.204	0.626	0.675	0.601
	Edge group	0.378	0.798	0.526	0.511
	Platform group	0.000	0.333	NC	NC
AT103	Overall localities	0.216	0.417	0.483	0.416
	Edge group	0.362	0.590	0.386	0.403
	Platform group	0.056	0.378	0.853	0.750

**Notes.**

H_*S*_ average gene diversity within localities H_*T*_ total gene diversity G_*ST*_ interlocality differentiation N_*ST*_ number of substitution types NC not calculated

**Table 5:** Results of neutrality tests and mismatch distribution analysis for the whole gene pool of *Triosteum pinnatifidum* based on cpDNA and AT103 sequence datasets.

		**Tajima’s** ***D*** **test**		**Fu’s** ***Fs*** **test**		**Mismatch distribution**			
**Sequences**	**Groups**	***D***	***p*** **value**	***Fs***	***p*** **value**	**SSD**	***p*** **value**	**HRI**	***p*** **value**
cp DNA	Overall	−1.25	0.10	−3.58	0.047	0.002	0.38	0.12	0.32
	Edge group	−0.82	0.26	−1.35	0.22	0.01	0.15	0.12	0.09
	Platform group	−0.63	0.21	−0.36	0.17	0.04	0.13	0.59	0.43
AT103	Overall	−0.77	0.27	−2.38	0.07	0.003	0.41	0.31	0.56
	Edge group	−0.23	0.37	0.30	0.50	0.009	0.19	0.19	0.42
	Platform group	0	1	−1.33	0.11	0.0006	0.36	0.52	0.65

**Notes.**

SSD sum of squared deviation under expansion model HRI Harpending’s raggedness index

**Figure 3:**
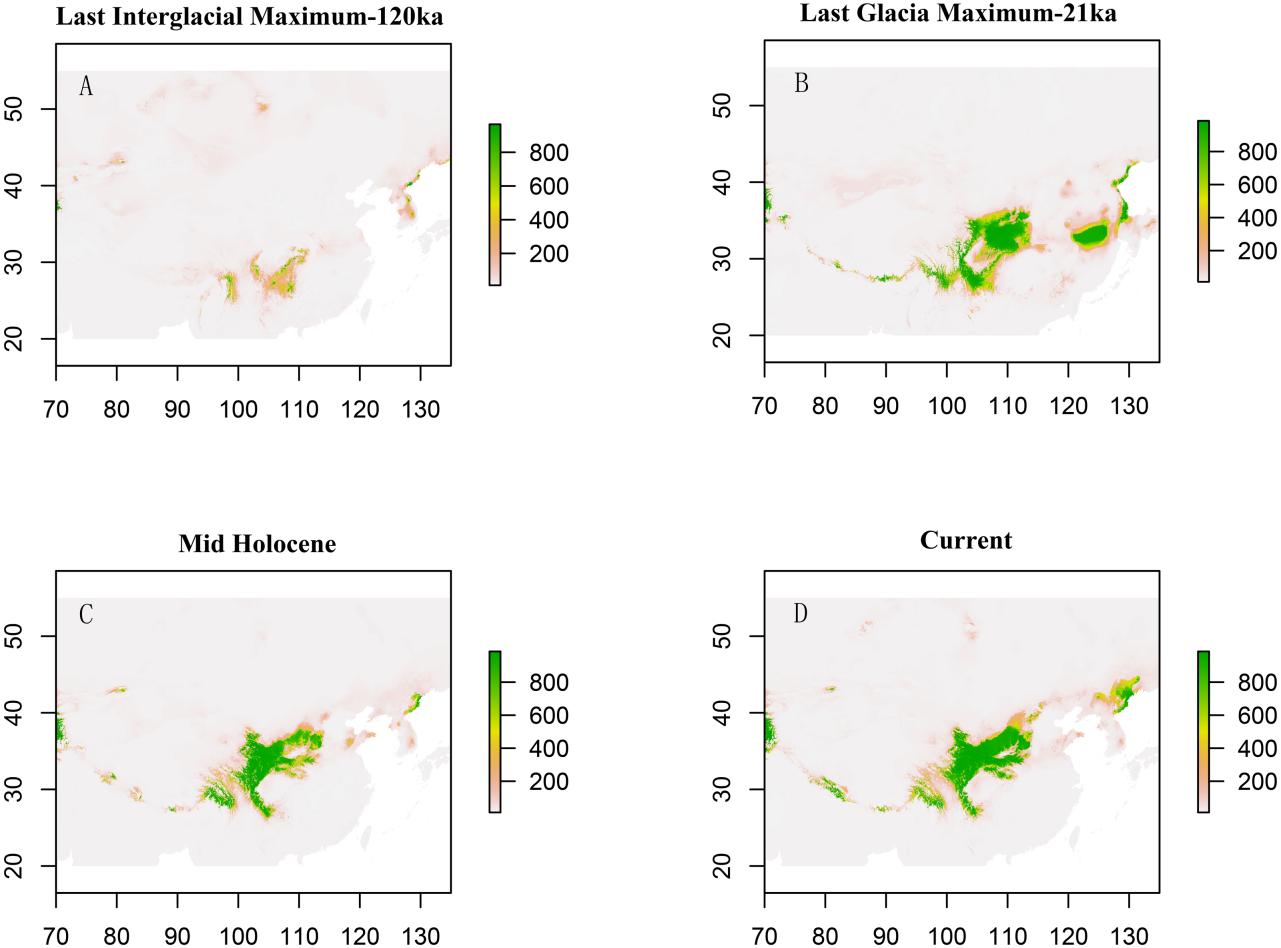
Estimated climatic niche models for *Triosteum pinnatifidum*. Maps are shown for each of the four periods tested. (A) Suitable areas for LIG. (B) Suitable areas for LGM. (C) Suitable areas for the Mid-Holocene. (D) Current suitable areas.

Paleodistribution modelling also supported the hypothesis of increased suitable habitat, mainly from the QTP to its periphery (Fig. 3). The Paleodistribution reconstruction showed that distinct suitable habitat expansions occurred in last interglacial maximum (LIG, 120 ka) up to the last glacial maximum (LGM, 21 ka) but went through partial reduction during the Mid-Holocene, and species from the Mid-Holocene to the present showed an expanding behavior that was not significant as it happened on a small range.

The result of bayesian skyline plot analyse indicated that *T. pinnatifidum* experienced three large population increases between 5–7 Ma, followed by a small decline about 2 Ma, and remained relatively stable for the rest of the time.

## Discussion

### Idiosyncratic patterns of seed and pollen-mediated gene flow

In this study, the cpDNA-concatenated sequences revealed higher levels of population differentiation than the nuclear DNA low copy sequences on both the overall and group levels (*F*_ST_ = 0.654 *vs.* 0.5763 *vs.* 1.00 for cpDNA, *F*_ST_ = 0.398 *vs.* 0.2411 *vs.* 0.6606 for AT103 of the overall distribution, Edge group, and Platform group respectively). This may indicate that pollen gene flow has a widespread influence on the *T. pinnatifidum* population. A study by Ennos (1994) suggested that when pollen or seed migration occurred, population differentiation of the maternal genetic markers was greater than that of nuclear markers. The gene flow for overall localities was 0.132 (*N*_m_ < 1), indicating divergence between localities (Millar & Libby, 1991).

Similarly, the study of *Quercus aquifolioides* revealed a stark contrast in the population differences between combined survey of chloroplast and nuclear DNA (*F*_ST_: cpDNA = 0.98, nSSR = 0.07). This was regarded as evidence for extremely limited seed gene flow but extensive pollen gene flow (Du et al., 2017). Such a pattern was also found in *Corydalis hendersonii*, and the different inheritance modes and dispersal style among localities, as well as the fast substitution rate and concerted evolution of nuclear sequences were suggested as possible explanations (Li et al., 2020). Gao et al. (2016) also reported a conflict in the phylogeographic patterns of *Rhodiola chrysanthemifolia* (Crassulaceae) as revealed by chloroplast and nuclear markers; however, population differentiation was higher for ITS sequences (*F*_ST_ = 0.9627) than for cpDNA (*F*_ST_ = 0.6368) markers. Seed migration that occurred among localities that could homogenize maternally inherited genetic variation and inbreeding in localities of *R. chrysanthemifolia* were interpreted as the two causes.

As described above, *T. pinnatifidum* seed has limited germination rates and lower dispersal efficiency than pollen. In conclusion, the conflicting patterns of population differentiation in *T. pinnatifidum* could be due to matrilineal inheritance of cpDNA markers in the case of restricted seed migration. Our results demonstrated that different sources of genetic variation for DNA markers could provide divergent molecular information. Thus, the joint application of plastid and nuclear DNA markers could improve the research accuracy of genetic variation and population structure. Additionally, more low- or single-copy nuclear genes are needed to promote the dataset in future studies.

### Molecular variance and population differentiation

Low-level within-locality diversity (*H*_S_) was detected for the two datasets (*H*_S_ = 0.204 for cpDNA and *H*_S_ = 0.216 for AT103, Table 4). Former genetic diversity and phylogeographic structures of *T. pinnatifidum* only based on cpDNA *rbc*L-*acc*D showed similar results (Liu et al., 2018a; Liu et al., 2018b). The total genetic diversity (*H*_T_) of cpDNA was higher than that of AT103 in this species (*H*_T_ = 0.626 for cpDNA and *H*_T_ = 0.417 for AT103, Table 4). The harmonic analysis results for cpDNA and nuclear DNA showed that *H*_T_ was higher than *H*_S_, suggesting that the genetic diversity within the locality was lower than the total when the haplotypes were widely distributed.

However, compared with other studied alpine species occurring in the QTP and adjoining regions, the genetic diversity of *T. pinnatifidum* was relatively low (*H*_T_ = 0.820 and *H*_S_ = 0.555, Khan et al., 2014) *H*_T_ = 0.950, *H*_S_ = 0.571, (Gao et al., 2012); *H*_T_ = 0.937 and *H*_S_ = 0.411 for cpDNA; *H*_T_ = 0.970 and *H*_S_ = 0.424 for ITS, (Gao et al., 2016). Few species have shown similar results (*H*_T_ = 0.558 and *H*_S_ = 0.142, (Chen et al., 2008); *H*_T_ = 0.316, *H*_S_ = 0.111, Jian, Tang & Sun, 2015). Even the genetic diversity and population structure of species could be influenced by life history characteristics (*e.g.*, life cycle, seed and pollen dispersal, and pollination form) and environmental factors (*e.g.*, geographical range, climate, and topography) (Hamrick, Linhart & Mitton, 1979; Loveless & Hamrick, 1984; Nybom, 2004; Zhang, Volis & Sun, 2010). The genetic structure revealed here for *T. pinnatifidum* may result mainly from the high frequency of common haplotypes (H2 for cpDNA and S2 for AT103) throughout the distribution (Table 1). Furthermore, a positive association between population size and genetic variation was supported for natural plant populations (Ellstrand & Elam, 1993), and different sample sizes could generate a different genetic diversity (Wei & Ashman, 2018). Due to the limited number of samples in some localities in current analyses, the comparability of genetic parameters among sampling locations needs further investigation with more detailed sampling in future researches.

### Demographic history and glacial refugia of *T. pinnatifidum*

In several former studies, the “out of QTP” hypothesis has been demonstrated, suggesting that the QTP is the center of origin for many species (Weigold, 2005; Jia et al., 2012; Wang et al., 2014a; Wang et al., 2014b; Pisano et al., 2015; Ebersbach et al., 2017; Mosbrugger et al., 2018). However, some species were dispersed into the QTP region. In these studies, rare mutations and an excess of star-like gene networks indicated demographic expansions of the populations (Posada, Crandall & Templeton, 2000; Gao et al., 2016; Liu et al., 2018a; Liu et al., 2018b). The decline in haplotype variations of *T. pinnatifidum* from the plateau edge and adjoining highlands to the platform of the QTP suggested a marked founder effect in the relatively recent past (cpDNA: *H*_T_ = 0.798 for the Edge group vs. *H*_T_ = 0.333 for the Platform group, AT103: *H*_T_ = 0.590 for the Edge group *vs. H*_T_ = 0.378 for the Platform group; Gugerli et al., 2001; Meng et al., 2007).

The dominant haplotypes H2 and S2 were contained in several localities from the plateau edge and adjacent highlands as well as in most plateau platform localities. Thus, our survey supports the “into the QTP” hypothesis that QTP platform localities were colonized from either a single refugium or from several refugia localities (*e.g.*, P8/P12) that were more widely distributed along the edge of the QTP platform. This is similar to the third hypothesis explaining species range shifts on the QTP, as described previously. For the founder effect, after glaciation or other conditions, the genetic diversity of localities in the glacier refugia would be more abundant than that in later or recently occupied areas.

The phylogeographic structure resolved in *T. pinnatifidum* was similar to those reported for *Frangula alnus* (Hampe et al., 2003), *Juniperus przewalskii* (Zhang et al., 2005), *Picea crassifolia* (Meng et al., 2007), and *Pedicularis longiflora* (Yang et al., 2008). These species also showed a higher level of population differentiation in their refuge area but almost complete genetic homogeneity in recolonized regions. Many previous studies reported that during the LGM or former glaciations, the cooler and drier climates may have forced this species to retreat to the lower and warmer plateau edge and/or adjacent highlands. In contrast, during the post/inter-glacial periods, a suitable environment and climate promoted the recolonization of this alpine herb to the plateau platform (Gao et al., 2016; Khan et al., 2018b). In contrast, *Triosteum pinnatifidum* appears to have experienced suitable habitat expansion from LIG to LGM.

The paleodistribution reconstruction showed an overall demographic expansion of suitable habitat: (i) distinct expansions occurred in the last interglacial maximum up to the last glacial maximum, (ii) a partial reduction occurred during the Mid-Holocene, and (iii) from the Mid-Holocene to the current period, the species has expanded, which is not significant as it has occurred on a small range. Range expansion in LGM or other cold periods has been reported for several alpine plant species distributed in the QTP and adjoining areas (Shimono et al., 2010; Liu et al., 2012; Fan et al., 2013; Li et al., 2013; Sun et al., 2014; Wei & Zhang, 2021). This pattern could be interpreted as follows: (1) the QTP was not covered by ice sheets during the last glacial period (Shi, 2002; Shimono et al., 2010); and (2) the moderately cold climate prevailed in LIG, which would provide opportunities for *T. pinnatifidum* to expand its range and continue suitable habitat expansion in lower elevations in the relatively cold LGM (Li et al., 2013; Wei & Zhang, 2021).

Similarly, the star-like network together with the unimodal distribution of mismatches (Fig. 2) based on the two datasets also support the presumption that *T. pinnatifidum* has experienced current demographic expansion. The mismatch distribution analysis with SSD and raggedness index values did not reject a sudden expansion model. Additionally, bayesian skyline plot analysis of *T. pinnatifidum* showed three large population increases between 5–7 Ma.

In certain plateau edge and adjacent highland localities, there was an absence of dominant haplotypes. In particular, locality P2 (H7 for cpDNA, S1 for AT103) was fixed with no dominant haplotype for either the cpDNA or AT103 dataset. However, the star-like network of the cpDNA and AT103 haplotypes suggested that all branch haplotypes originated from the dominant haplotypes H2 and S2, respectively. The isolation created by the characteristic topography and environment generally leads to the reduction of genetic variation and conservation of private haplotypes. Thus, isolation could be the cause of the absence of dominant haplotypes in certain localities.

## Conclusions

The development and retreat of glaciers and the ecological environment have had a marked impact on the species distribution and genetic structure. Here, the genetic structure, diversity, and demography of an important medicinal herb species, *T. pinnatifidum*, was assessed for the first time. An integrated approach of paleodistribution reconstruction combined with genetic data was used. Disparate patterns of genetic diversity based on cpDNA and nuclear DNA markers were observed.

There was higher genetic variation based on the cpDNA in *T. pinnatifidum* localities than that based on nuclear DNA. This idiosyncratic pattern might be due to a high level of pollen-mediated gene flow and limited gene flow through seeds in the *T. pinnatifidum* locality. Our results further revealed that there may have been one or several refugia for this species during glaciation. The dispersal of *T. pinnatifidum* from the plateau edge and adjoining highland to the plateau platform was also represented. We refrained from making strong conclusions; however, our work provides a basis to substantiate the alternative hypothesis regarding range shift on the QTP and adjacent highlands.

## Supplemental Information

10.7717/peerj.12754/supp-1 Supplemental Information 1 Haplotypes of *Triosteum pinnatifidum*

10.7717/peerj.12754/supp-2 Supplemental Information 2 Correlation coefficients between the climate variables used for analyses Bio1-annual mean temperature (°), bio2-mean diurnal range [mean of monthly max temp-min temp] (° ), bio3-isothermality (BIO2/BIO7)( ×100), bio12-annual precipitation (mm), bio14-precipitation of driest month (mm), bio15-precipitation seasonality (mm)

10.7717/peerj.12754/supp-3 Supplemental Information 3 Bayesian skyline plot representing historical demography in effective population size of *T*. *pinnatifidum*

## Additional Information and Declarations

### Data Availability

The following information was supplied regarding data availability: All newly generated sequences from Triosteum pinnatifidum are available at GenBank: MH277369 –MH277382.
