# Supplementary figures and images for: Dispersal into the Qinghai–Tibet plateau: evidence from the genetic structure and demography of the alpine plant Triosteum pinnatifidum
Source: PeerJ. 2022 Feb 1;10:e12754. doi: 10.7717/peerj.12754 (PMC8815373; doi:10.7717/peerj.12754)

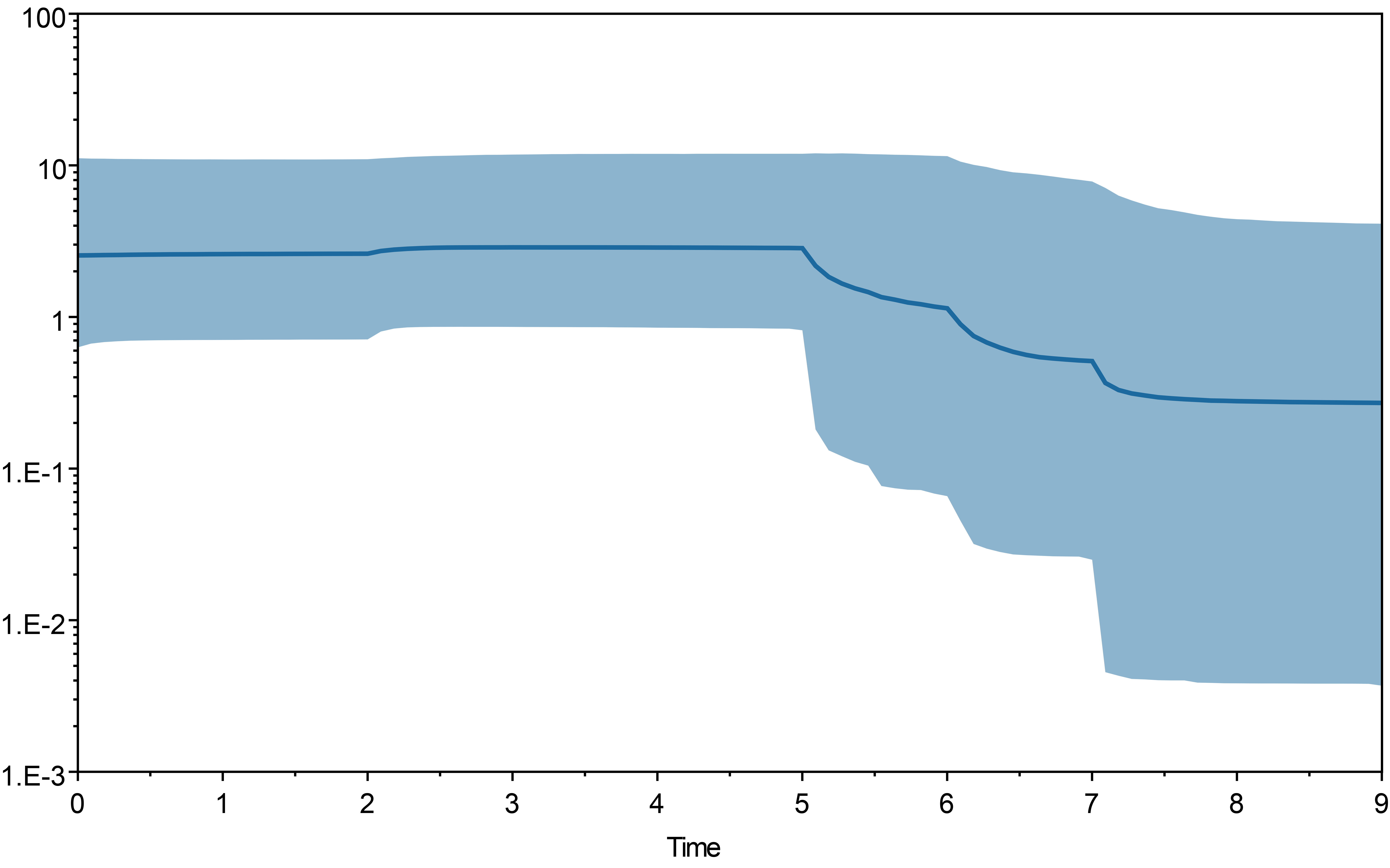

Supplement: Supplemental Information 3 [file peerj-10-12754-s003.jpg]
